# MFBCE: A Multi-Focal Bionic Compound Eye for Distance Measurement

**DOI:** 10.3390/s25092708

**Published:** 2025-04-24

**Authors:** Qiwei Liu, Xia Wang, Jiaan Xue, Shuaijun Lv, Ranfeng Wei

**Affiliations:** Key Laboratory of Photoelectronic Imaging Technology and System, Ministry of Education of China, Beijing Institute of Technology, Beijing 100081, China; 3220205076@bit.edu.cn (Q.L.); 3120185345@bit.edu.cn (J.X.); 3120220569@bit.edu.cn (S.L.); wtfbit@126.com (R.W.)

**Keywords:** compound eye, multi-focal, ranging, distance measurement

## Abstract

In response to the demand for small-size, high-precision, and real-time target distance measurement in platforms such as autonomous vehicles and drones, this paper investigates the multi-focal bionic compound eye (MFBCE) and its associated distance measurement algorithm. MFBCE was designed to integrate multiple lenses with different focal lengths and a CMOS array. Based on this system, a multi-eye distance measurement algorithm based on target detection was proposed. The algorithm derives the application of binocular distance measurement on cameras with different focal lengths, overcoming the limitation of traditional binocular algorithms that only work with identical cameras. By utilizing the multi-scale information obtained from multiple lenses with different focal lengths, the ranging accuracy of the MFBCE is improved. The telephoto lenses, with their narrow field of view, are beneficial for capturing detailed target information, while wide-angle lenses, with their larger field of view, are useful for acquiring information about the target’s environment. Experiments using the least squares method for ranging targets at 100 cm yielded a mean absolute error (MAE) of 1.05, approximately one-half of the binocular distance measurement algorithm. The proposed MFBCE demonstrates significant potential for applications in near-range obstacle avoidance, robotic grasping, and assisted driving.

## 1. Introduction

Distance measurement is a critical component of navigation and has a wide range of applications in fields such as criminal investigation, national defense, and transportation. Distance measurement methods can be broadly classified into two categories: active and passive. Active distance measurement methods involve the system actively emitting signals and analyzing the returning echoes to calculate the distance. These methods typically offer long measurement ranges, high accuracy, and strong penetration capabilities but come with high costs and safety concerns [1]. In contrast, passive distance measurement methods rely on image processing and do not require the system to emit signals. These methods are more cost-effective, concealed, and easy to operate, garnering increasing attention. Binocular distance measurement is a representative passive distance measurement [2,3] that simulates the human visual perception mechanism, reconstructing target distances based on triangulation. However, binocular distance measurement has several limitations, such as difficulties in balancing compact sizes with a wide field of view, restrictions to identical cameras, and significant measurement errors when the baseline is short. To address these issues, bionic compound eyes and multi-eye distance measurement algorithms have emerged as prominent research focuses both domestically and internationally in recent years.

Early bionic compound eyes often employed a microlens array [4], which featured small apertures, low resolutions, short baselines, and limited imaging distances, making them unsuitable for distance measurement applications. In recent years, multi-camera arrays [5] composed of multiple lenses and CMOS arrays have emerged as a new paradigm for compound eye systems. These systems offer high resolutions and long imaging distances, holding significant potential in the fields of public security [6], 3D sensing [7,8], medical imaging [9,10], and bionic navigation [11]. An ultra-thin high-performance monolithic camera array named Pelican Imaging Camera Array (PiCam) was presented by Venkataraman et al. [12], which has a huge potential application for mobile devices because it supports still images and videos, and it has low-light compatibility due to its small size. Afshari H et al. [13,14] designed a multi-camera system named the panoptic camera, which consists of a layered arrangement of thirty classical CMOS image sensors distributed over a hemisphere. Cao et al. [15] designed a spherical bionic artificial compound eye imaging system consisting of a spherical support and 37 sub-eye lenses. Carles G et al. [16] designed a multi-aperture image system consisting of 5 × 5 commercial low-cost cameras assembled into a plane array. Popovic et al. [17] designed a panoptic camera system consisting of five floors and 49 cameras. The 49 cameras are distributed on a sphere with a 30 cm diameter. Xue et al. [18] designed a bionic compound eye system based on a fiber faceplate. This system integrates nine lenses coupled with a CMOS camera featuring a fiber faceplate, enabling stereovision and motion target detection.

Moreover, compared to binocular distance measurement algorithms, the multi-eye distance measurement algorithms of bionic compound eyes utilize multi-scale information, achieving higher accuracy. Horisaki R et al. [19] proposed an effective method for three-dimensional information acquisition based on the thin observation module by bound optics (TOMBO). An image captured by the TOMBO system is composed of multiple images observed from several viewpoints. The distance (100 mm) between the TOMBO system and objects can be estimated using the parallax of the captured images. Lee et al. [20] developed a sparse-representation-based classification algorithm to estimate object depths in the COMPU-EYE imaging system. In their experiments, four characters were located at three different distances (108 mm, 109 mm, and 112 mm) from the compound eye. The proposed system with this depth estimation method can provide a depth map of the object with a 1 mm depth resolution. Yang et al. [21] proposed a positioning algorithm based on an optical fiber panel compound eye. Experiments were conducted to measure targets at distances ranging from 1 m to 1.6 m, with an average ranging error of 3.7%. Liu et al. [22] demonstrate the 3D measurement using a curved compound-eye camera. The experimental results show that the working range for 3D measurement can cover the whole FOV of 98°, and the working distance can be as long as 3.2 m, with a measurement error of no more than 5%. Oh W et al. [23] proposed a transformer-based neural network for eye-wise depth estimation, which is suitable for the compound eye image. The experiments were conducted within a 4.5 m range. The proposed method achieved a mean absolute error of 0.20 m on the GAZEBO dataset and 0.29 m on the Matterport3D dataset. Wang et al. [24] proposed a simulation method to evaluate the imaging and ranging performance of the designed infrared compound eye. The distance measurement error within 1 m is approximately 0.2 m.

Currently, bionic compound eyes demonstrate excellent performance in high-resolution imaging and wide-field detection. However, they struggle to simultaneously meet compact size, light weight, and clear imaging requirements [25,26], limiting their applicability to platforms with spatial and computational constraints, such as small unmanned vehicles and drones. Moreover, deep-learning-based multi-eye ranging methods are often constrained by hardware requirements. While binocular ranging can realize relatively accurate target distance estimation, it is typically limited to identical camera setups. Additionally, its accuracy is restricted by the baseline distance between the two cameras and requires prior calibration. When the baseline is short, the ranging precision is significantly reduced. To address these limitations, it is essential to optimize the structural design of bionic compound eye systems and their distance measurement algorithms. This optimization aims to enable high-precision distance measurement under conditions of compact size, short baseline, and varying focal lengths.

This paper addresses the challenges associated with the system architecture and distance measurement algorithms of existing bionic compound eyes by developing a multi-focal bionic compound eye (MFBCE) for distance measurement.

The main contributions of this paper are as follows:

- We Design an MFBCE. This system integrates an onboard core processing unit to handle image data, thereby reducing reliance on GPUs. This design significantly decreases the weight and size of the bionic compound eye (Section 2).
- We propose a multi-eye distance measurement algorithm. By utilizing multiple lenses with different focal lengths to capture multi-scale target images and deriving the intrinsic relationships between these images, the algorithm overcomes the limitations of binocular methods, which require identical cameras and prior calibration. This approach improves the ranging accuracy of the system (Section 3).
- We conducted an analysis of distance measurement errors. The MFBCE, combined with the multi-eye distance measurement algorithm, was used to conduct experiments for error analysis (Section 4).

The MFBCE proposed in this paper is characterized by its compact structure, light weight, and high imaging accuracy. When combined with the multi-eye distance measurement algorithm presented, it enables high-precision distance measurement for near-range targets. The system achieves a mean absolute error of 0.54 for targets within the range of 90 cm to 120 cm. This system holds significant potential for applications in areas such as emergency obstacle avoidance in unmanned vehicles, robot grasping, and advanced driver assistance systems [27,28,29].

## 2. Design of MFBCE

### 2.1. Structure of MFBCE

**Figure 1:**
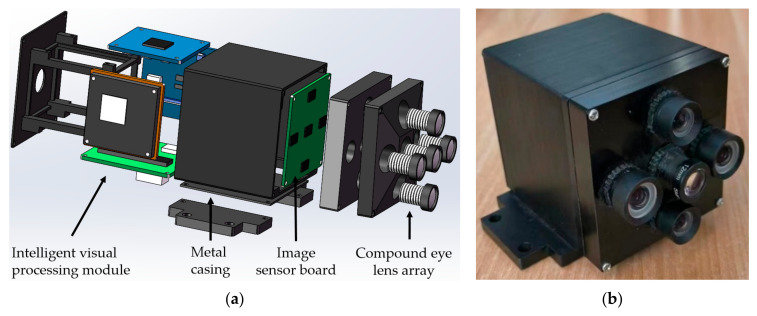
Structure of the multi-focal bionic compound eye (MFBCE). (**a**) Design diagram; (**b**) real object image.

The structure of MFBCE includes the compound eye lens array, image sensor board, intelligent visual processing module, and metal casing. The compound eye lens array consists of lenses with focal lengths of 6 mm, 8 mm, and 12 mm. The left and right optical paths use 6 mm focal length lenses, the up and down optical paths use 8 mm focal length lenses, and the central optical path uses a 12 mm focal length lens. The image sensor board is composed of five CMOS image sensors, with each sensor featuring a pixel size of 2.8 µm × 2.8 µm and a resolution of 1920 × 1080. The intelligent visual processing module includes RV1126 and RV1106 intelligent vision chips, as well as an image signal transmission board. The target distance measurement algorithm is deployed on the RV1126 chip. This module realizes the processing of visual signals and the interaction with external information. The metal casing is made of aluminum alloy, which is lightweight and corrosion-resistant. It serves to connect and secure the components while protecting the internal structure. The parameters of the MFBCE are presented in Table 1, with the structure shown in Figure 1.

### 2.2. Principle of MFBCE

**Figure 2:**
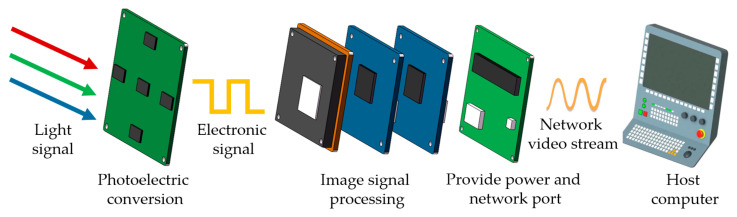
Principle of the MFBCE.

The principle of MFBCE is shown in Figure 2. Light signals containing both target and background information are projected onto the image sensor board through the compound eye lens array. The light signals are then converted into electronic signals through photoelectric conversion on the image sensor board, generating the raw image. The raw image is subsequently processed by the RV1126 and RV1106 intelligent vision chips, which feature image signal processing (ISP) functions. These chips convert the raw image into a format that aligns with human visual perception. Additionally, the RV1126 and RV1106 chips support video-encoding inputs and outputs. The processed image is input into a deep learning model via the video input (VI) channel for object detection. After detection, the image is encoded through the video encoder (VENC) to generate a network video stream based on a real-time streaming protocol (RTSP). Finally, the network video stream is transmitted to the host computer through the image signal transmission board. The real-time detection and distance measurement of the target can be observed using a video player.

### 2.3. Field of View of MFBCE

The lens array of the MFBCE consists of lenses with focal lengths of 6 mm, 8 mm, and 12 mm. Long-focus lenses provide high imaging precision but have a narrow field of view, allowing for the capture of fine target details and texture information. In contrast, short-focus lenses offer a wide field of view but lower imaging precision, enabling large-area target detection when combined with object detection algorithms. The multi-focal bionic compound-eye system integrates the advantages of both—combining the high imaging precision of telephoto lenses with the wide field of view of short-focus lenses—thereby enabling multi-scale target observation. The rich image data obtained through this approach can be applied across various fields.

The field of view (FOV) of the MFBCE includes the diagonal field of view (DFOV), horizontal field of view (HFOV), and vertical field of view angle (VFOV). The FOV can be calculated based on the pixel size, resolution, and focal length. The results are shown in Table 2.

**Figure 3:**
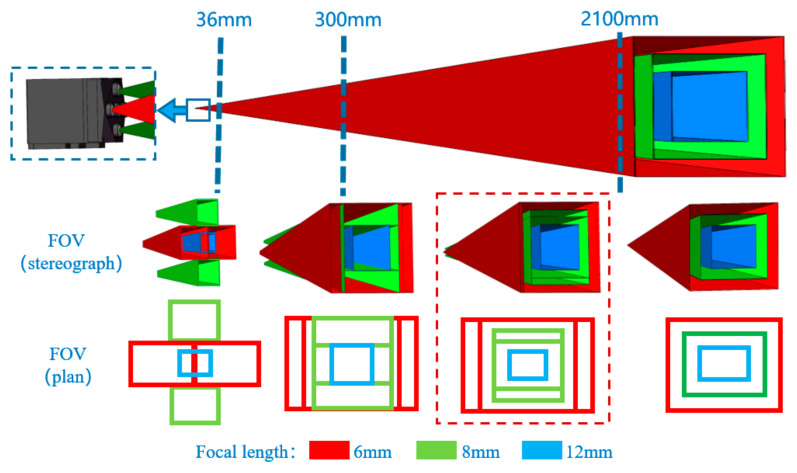
Field of view (FOV) of MFBCE.

We modeled the FOV of MFBCE, as shown in Figure 3. In the figure, the red regions represent the FOV of the 6 mm focal length lenses, the green regions represent the 8 mm focal length lenses, and the blue regions represent the 12 mm focal length lenses. Due to the parallax between different lenses, different FOV distributions were observed at various imaging distances:

- 36 mm: The FOV of four lenses at the top, bottom, left, and right intersect at this distance. Distances less than 36 mm create a blind area.
- 300 mm: The FOV of four lenses covers the center lens’s FOV at this distance.
- 300–2100 mm: The FOVs of the five lenses exhibit significant overlap, with noticeable differences caused by the parallax.
- Greater than 2100 mm: The overlap between the FOVs of the 6 mm focal length lenses reaches 98%, while the overlap between the 8 mm focal length lenses is 95%. The effect of parallax becomes smaller, and the system can be approximated as a coaxial system.

**Figure 4:**
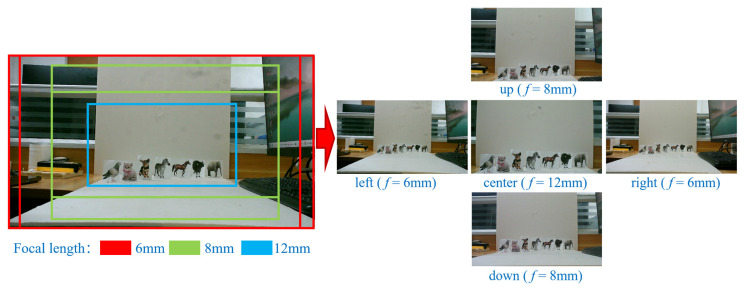
Real imaging field of view of MFBCE.

Based on the above analysis, it can be concluded that when the imaging distance of MFBCE is 300–2100 mm, the target observed by the central lens can simultaneously be captured by the surrounding four lenses (Figure 4). Significant differences in the FOV arise due to the parallax. In this range, the five lenses can collectively observe the target in the central region. Subsequently, by analyzing the differences in the target projections on the imaging planes of the different lenses, the high-precision distance measurement of the target can be realized. Furthermore, increasing the distance between lenses can significantly enhance the parallax, thus extending the system’s measurement range. However, a larger distance between lenses also increases the system’s volume and introduces larger visual blind areas. In the practical use of the MFBCE, lenses with different focal lengths can be swapped based on the measurement distance requirements. Telephoto lenses are selected for distant and small targets to improve accuracy, while wide-angle lenses are chosen for close-range and large targets to avoid blind spots and ensure that the target remains within the field of view.

## 3. MFBCE Distance Measurement Algorithm

**Figure 5:**
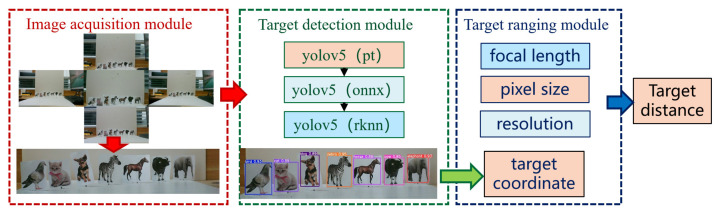
MFBCE distance measurement algorithm.

Currently, binocular distance measurement algorithms require identical lenses and cameras, as well as pre-calibrated camera parameters, which severely limit the universality of compound eyes. In contrast, depth estimation algorithms based on deep learning face challenges related to low accuracy. To address these issues, this paper proposes a multi-eye distance measurement algorithm based on target detection. By deriving the intrinsic relationship between images captured by lenses with different focal lengths, the proposed method overcomes the limitations of traditional binocular algorithms that require identical cameras and pre-calibration. Furthermore, the algorithm achieves higher measurement accuracy for specific targets through target detection compared to global depth estimation. Additionally, by converting the yolov5.pt model and deploying it on the RV1126 vision chip, the algorithm eliminates the reliance on computers. The process of the algorithm is shown in Figure 5.

The MFBCE distance measurement algorithm consists of three modules: image acquisition module, target detection module, and target ranging module. First, five images containing the target are captured by MFBCE. These images are then input into the intelligent vision chips of the MFBCE for target detection, where the target’s coordinates and category information are extracted. Finally, by incorporating camera parameters such as the pixel size, resolution, lens’s focal length, and distance between lenses, the target’s distance is derived through a set of equations:

- Image acquisition module: MFBCE captures the target simultaneously using five lenses with different focal lengths. The use of lenses with varying focal lengths allows for the acquisition of multi-scale information, which provides a solid foundation for target detection and ranging.
- Target detection module: The five acquired images are input into the intelligent vision chip of the MFBCE for target detection. In this study, we employ the YOLOv5 target detection algorithm [30,31]. Since traditional PyTorch models are not compatible with the RV1126 chip, the YOLOv5 model must be converted. First, the PyTorch model is converted into the ONNX (open neural network exchange), and then, the ONNX is further converted into the RKNN (rockchip neural network). RKNN is the deep learning neural network used by the RV1126 chip.
- Target ranging module: Through the image acquisition and target detection modules, the target bounding box and coordinates are obtained. Then, by integrating information such as the CMOS pixel size, camera resolution, lens’s focal length, and distance between lenses, the target’s distance can be calculated through theoretical derivation. This process is elaborated in detail in the following sections.

### 3.1. Monocular Vision Distance Measurement Model

**Figure 6:**
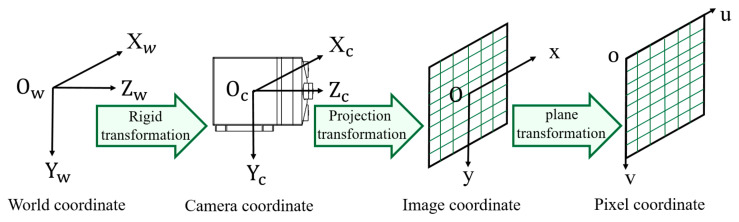
Transformation between four coordinate systems.

To obtain 3D distance information from a 2D image, it is necessary to model the camera and analyze the corresponding relationship between a point in the physical space and its projection onto the camera image based on the camera imaging principle [32,33]. This involves the transformation between four coordinate systems: the world coordinate system (O_w_-X_w_Y_w_Z_w_), camera coordinate system (O_c_-X_c_Y_c_Z_c_), image coordinate system (O-xy), and pixel coordinate system (o-uv). The relationship between these coordinate systems is illustrated in Figure 6. Let the coordinates of a point P be denoted as (*X_w_*, *Y_w_*, *Z_w_*), (*X_c_*, *Y_c_*, *Z_c_*), (*x*, *y*), and (*u*, *v*) in the respective coordinate systems.

The transformation from the world coordinate system to the camera coordinate system is a rigid transformation, meaning that the camera undergoes no deformation but only rotation and translation, as shown in Equation (1):

(1) $$\left[\begin{array}{c} X_{c}\\ Y_{c}\\ Z_{c} \end{array}\right]=R\left[\begin{array}{c} X_{w}\\ Y_{w}\\ Z_{w} \end{array}\right]+T\Rightarrow \left[\begin{array}{c} \begin{array}{c} X_{c}\\ Y_{c}\\ Z_{c}\\ 1 \end{array} \end{array}\right]=\left[\begin{array}{cc} R & T\\ 0 & 1 \end{array}\right]\left[\begin{array}{c} \begin{array}{c} X_{w}\\ Y_{w}\\ Z_{w}\\ 1 \end{array} \end{array}\right]$$

where *R* is a 3 × 3 rotation matrix, and *T* is a 3 × 1 translation vector.

The transformation from the camera coordinate system to the image coordinate system is a perspective projection, which, based on the pinhole imaging principle, projects real-world 3D objects onto a 2D image, as shown in Equation (2):

(2) $$Z_{c}\left[\begin{array}{c} x\\ y\\ 1 \end{array}\right]=\left[\begin{array}{ccc} f & 0 & 0\\ 0 & f & 0\\ 0 & 0 & 1 \end{array}\right]\left[\begin{array}{c} X_{c}\\ Y_{c}\\ Z_{c} \end{array}\right]$$

where *f* represents the focal length of the camera lens.

The transformation from the image coordinate system to the pixel coordinate system is a plane transformation, where the units of length are converted into pixel units, as shown in Equation (3):

(3) uv1=1dx0u001dyv0001xy1

where *d_x_* and *d_y_* represent the physical length of a single pixel in the x and y directions of the camera’s sensor, respectively, which corresponds to the CMOS pixel size. u0 and v0 are the center point coordinates of the pixel coordinate system.

By combining Equations (1)–(3), the relationship between the pixel coordinate system and the world coordinate system can be derived, as shown in Equation (4):

(4) Zcuv1=fdx0u000fdyv000010RT01XwYwZw1

where the matrix containing $f/d_{x}$, $f/d_{y}$, $u_{0}$, and $v_{0}$ is the camera’s intrinsic matrix, which is related to the camera’s internal parameters. The matrix containing *R* and *T* is the camera’s extrinsic matrix, which reflects the camera’s position in the world coordinate system.

In this paper, the distance to the target relative to the camera needs to be calculated. In this case, the camera coordinate system coincides with the world coordinate system (*X_w_* = *X_c_* = *X*, *Y_w_* = *Y_c_* = *Y*, *Z_w_* = *Z_c_* = *Z*). With the CMOS parameter $d_{x}=d_{y}=d$, Equation (4) can be simplified into the following:

(5) uv1=fd0u00fdv0001XZYZ1

Equation (6) can be derived as follows from Equation (5):

(6) $$u=\frac{fX}{dZ}+u_{0},v=\frac{fY}{dZ}+v_{0}$$

The size of the target in the pixel coordinate system can be correlated with its actual size using Equation (6), as shown in Equation (7):

(7) $$w=u_{R}-u_{L}=(\frac{fX_{R}}{dZ}+u_{0})-(\frac{fX_{L}}{dZ}+u_{0})=\frac{f(X_{R}-X_{L})}{dZ}=\frac{fW}{dZ}$$

where *u_R_* and *u_L_* represent the left and right horizontal coordinates of the target’s projection in the image bounding box, *X_R_* and *X_L_* represent the left and right horizontal coordinates of the target in the real world, and w represents the width of the target in the pixel coordinate system, while W denotes the actual width of the target.

From Equation (7), it can be observed that in the monocular vision model, the distance between the target and the camera can be determined based on the actual size of the target, its size in the pixel coordinate system, and the camera’s intrinsic parameters (focal length and pixel size).

### 3.2. Binocular Vision Distance Measurement Model

In practical applications, the camera’s intrinsic parameters (focal length and pixel size) can be obtained from system design specifications or product manuals. The target’s position and size in the pixel coordinate system can be extracted from the bounding box provided by the target detection algorithm. However, the actual size of the target is often unknown. To address this problem, at least two cameras are required to simultaneously observe the target, utilizing the parallax to infer missing scale information.

When cameras *i* and *j*, positioned at different locations, capture images of a target with an actual width of W, the following is obtained:

(8) $$W=\frac{d_{i}w_{i}Z_{i}}{f_{i}}=\frac{d_{j}w_{j}Z_{j}}{f_{j}}$$

where *d_i_* and *d_j_* represent the pixel sizes of cameras *i* and *j*, *f_i_* and *f_j_* denote the focal length of the lenses in cameras *i* and *j*, *w_i_* and *w_j_* correspond to the target’s projected width in the pixel coordinate systems of cameras *i* and *j*, and *Z_i_* and *Z_j_* represent the distances between the target and cameras *i* and *j*, respectively.

Similarly, when cameras *i* and *j*, positioned at different locations, capture images of a target with an actual height of H, the following is obtained:

(9) $$H=\frac{d_{i}h_{i}Z_{i}}{f_{i}}=\frac{d_{j}h_{j}Z_{j}}{f_{j}}$$

where *h_i_* and *h_j_* correspond to the target’s projected height in the pixel coordinate systems of cameras *i* and *j*.

**Figure 7:**
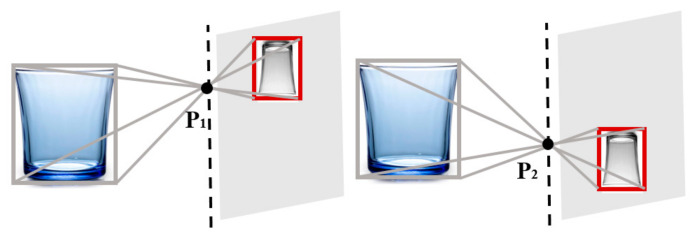
Target projection on different pixel coordinate systems.

The different positions of the cameras result in variations in the size and location of the target projection on the pixel coordinate systems, as illustrated in Figure 7. Taking into account the influence of the camera’s intrinsic parameters, the target distance can be determined using the camera parameters, inter-camera distance, and projection position. The following section presents the detailed derivation of the corresponding formulas.

Equation (10) can be derived as follows from Equation (8):

(10) $$Z_{j}=Z_{i}(\frac{f_{j}d_{i}w_{i}}{f_{i}d_{j}w_{j}})$$

By substituting Equation (10) into Equation (6), the relationship between the target’s size in the pixel coordinate system and its actual size can be established:

(11) $$u_{j}=\frac{f_{j}X_{j}}{d_{j}Z_{j}}+u_{j,0}=\frac{f_{j}X_{j}}{d_{j}Z_{i}(\frac{f_{j}d_{i}w_{i}}{f_{i}d_{j}w_{j}})}+u_{j,0}=(\frac{w_{j}}{w_{i}})\frac{f_{i}X_{j}}{d_{i}Z_{i}}+u_{j,0}$$

(12) $$u_{i}=\frac{f_{i}X_{i}}{d_{i}Z_{i}}+u_{i,0}$$

(13) $$(u_{j}-u_{j,0})(\frac{w_{i}}{w_{j}})-(u_{i}-u_{i,0})=\frac{f_{i}(X_{j}-X_{i})}{d_{i}Z_{i}}$$

where *u_j_* and *u_i_* represent the horizontal coordinates of the center of the bounding box observed by two cameras, while *u_j_*_,0_ and *u_i_*_,0_ represent the coordinates of the center of the pixel coordinate systems. The *X_i_* and *X_j_* positions of a static target depend only on the positions of the cameras and are given by the following:

(14) $$X_{j}-X_{i}=\Delta X$$

where Δ*X* is known as the baseline, which is the lateral distance between the two cameras. By combining Equations (13) and (14), the following can be obtained:

(15) $$Z_{i}=\frac{f_{i}\Delta X}{d_{i}(u_{j}-u_{j,0})(\frac{w_{i}}{w_{j}})-d_{i}(u_{i}-u_{i,0})}$$

Similarly, for cameras distributed parallel to the *Y*-axis, the following is the case:

(16) $$Z_{i}=\frac{f_{i}\Delta Y}{d_{i}(v_{j}-v_{j,0})(\frac{h_{i}}{h_{j}})-d_{i}(v_{i}-v_{i,0})}$$

where Δ*Y* is also known as the baseline, which is the longitudinal distance between the two cameras.

From Equations (8) and (9), it can be observed that when the cameras are at the same depth ($Z_{i}=Z_{j}$),

(17) $$\frac{w_{i}}{w_{j}}=\frac{h_{i}}{h_{j}}=\frac{d_{j}f_{i}}{d_{i}f_{j}}$$

It can be inferred that when different cameras capture the same target at the same depth, the ratio of the target’s projected size is solely dependent on the camera’s parameters. When the camera parameters are identical, the size of the target’s projection will also be the same.

Substituting Equation (17) into Equations (15) and (16), the following is obtained:

(18) $$Z_{i}=\frac{\Delta X}{\left(u_{j}-u_{j,0})(\frac{d_{j}}{f_{j}})-(u_{i}-u_{i,0})(\frac{d_{i}}{f_{i}}\right)}=\frac{\Delta Y}{\left(v_{j}-v_{j,0})(\frac{d_{j}}{f_{j}})-(v_{i}-v_{i,0})(\frac{d_{i}}{f_{i}}\right)}$$

From Equation (18), the target’s distance can be determined based on the baseline (Δ*X*, Δ*Y*), camera intrinsic parameters $\left(d_{i},d_{j},f_{i},f_{j}\right)$, the coordinates of the target’s bounding box center $\left(u_{i},u_{j},v_{i},v_{j}\right)$, and the center coordinates of the camera’s pixel coordinate system $\left(u_{i,0},u_{j,0},v_{i,0},v_{j,0}\right)$.

The baseline, camera intrinsic parameters, and coordinates of the center of the camera pixel coordinate system can be obtained from the system design. The coordinates of the target’s bounding box center can be obtained through the target detection algorithm. This algorithm addresses the scale ambiguity issue in the monocular vision model using binocular disparity and enables binocular ranging with cameras of different parameters (focal length, pixel size, and pixel array) without the need for pre-calibration.

### 3.3. Multi-Eye Vision Distance Measurement Model

The preceding discussion considers only the case of two cameras. In practical applications, errors in camera installation and target detection are inevitable. Therefore, we integrate the results from *n* cameras to obtain a more accurate distance calculation. Equation (19) can be derived as follows from Equation (18):

(19) $$\left[\begin{array}{c} \left(u_{j}-u_{j,0})(\frac{d_{j}}{f_{j}})-(u_{i}-u_{i,0})(\frac{d_{i}}{f_{i}}\right)\\ \left(v_{j}-v_{j,0})(\frac{d_{j}}{f_{j}})-(v_{i}-v_{i,0})(\frac{d_{i}}{f_{i}}\right) \end{array}\right]Z_{i}=\left[\begin{array}{c} \Delta X\\ \Delta Y \end{array}\right]$$

At this time, let the following be the case:

(20) $$\left[\begin{array}{c} \left(u_{j}-u_{j,0})(\frac{d_{j}}{f_{j}})-(u_{i}-u_{i,0})(\frac{d_{i}}{f_{i}}\right)\\ \left(v_{j}-v_{j,0})(\frac{d_{j}}{f_{j}})-(v_{i}-v_{i,0})(\frac{d_{i}}{f_{i}}\right) \end{array}\right]=\left[\begin{array}{c} \Delta x\\ \Delta y \end{array}\right]$$

By integrating the results from *n* observation points, we obtain the following:

(21) $${\left[\Delta x_{1},\Delta y_{1},\Delta x_{2},\Delta y_{2},\cdots ,\Delta x_{n},\Delta y_{n}\right]}^{T}\;Z_{i}={\left[\Delta X_{1},\Delta Y_{1},\Delta X_{2},\Delta Y_{2},\cdots ,\Delta X_{n},\Delta Y_{n}\right]}^{T}$$

where Δ*x* and Δ*y* represent the weighted pixel differences in the *X*-axis and *Y*-axis directions, respectively, between the two observation points. After the target is imaged, Δ*x* and Δ*y* can be calculated based on the camera’s intrinsic parameters, the coordinates of the target bounding box center, and the center coordinates of the camera’s pixel coordinate system. Additionally, Δ*X* and Δ*Y* denote the baseline, which is the distance between the two cameras.

To calculate a more accurate distance, the results observed by *n* cameras can be integrated. The distance can be calculated using the following method based on Equation (21):

Arithmetic mean (AM):

(22) $$Z_{\mathrm{AM}}=\frac{1}{n}\sum_{i=1}^{n}Z_{i}$$

Root mean square (RMS):

(23) $$Z_{RMS}=\sqrt{\frac{1}{n}\sum_{i=1}^{n}{\left(Z_{i}\right)}^{2}}$$

Least squares (LS):

(24) $$Z\to \min\left\{SSD(Z)\right\},SSD=\sum_{i=1}^{n}{\left(Z\Delta x_{i}-\Delta X_{i}\right)}^{2}+\sum_{i=1}^{n}{\left(Z\Delta y_{i}-\Delta Y_{i}\right)}^{2}$$

By varying the value of *Z*, *SSD* is calculated for different *Z* values. The specific distance *Z* that minimizes the *SSD* can be determined using the least squares method, and this *Z* is taken as the final distance calculation result.

Multi-eye distance measurement is subject to errors, with the primary causes being similar to those in binocular distance measurements. Specifically, ranging errors stem from mechanical inaccuracies introduced during the system’s assembly process and errors in object detection. Mechanical errors are inevitable during assembly and can affect the overall ranging precision. Our proposed algorithm is based on object detection, where higher object detection accuracies lead to improved ranging precision. Intersection over union (IoU) is a key metric for evaluating object detection algorithms, as it measures the ratio of the intersection area to the union area between the predicted bounding box and the ground truth. When the IoU value is low, the deviation between the predicted and actual bounding boxes increases, resulting in the greater displacement of the selected bounding box center coordinates from the ground truth, which in turn increases ranging errors.

Letting the error be denoted as *δ*, *Z*_pred_ can be calculated as follows:

(25) $$\begin{array}{ll} Z_{\mathrm{pred}} & =\frac{\Delta X}{\left(u_{j}-u_{j,0}+\delta_{1})(\frac{d_{j}}{f_{j}})-(u_{i}-u_{i,0}+\delta_{2})(\frac{d_{i}}{f_{i}}\right)}\\ & =\frac{\Delta Y}{\left(v_{j}-v_{j,0}+\delta_{3})(\frac{d_{j}}{f_{j}})-(v_{i}-v_{i,0}+\delta_{4})(\frac{d_{i}}{f_{i}}\right)} \end{array}$$

As derived from Equation (25), in practical applications, the ranging accuracy can be improved by increasing the baseline and using telephoto lenses to reduce the impact of the error *δ*.

## 4. Experimental Results

**Figure 8:**
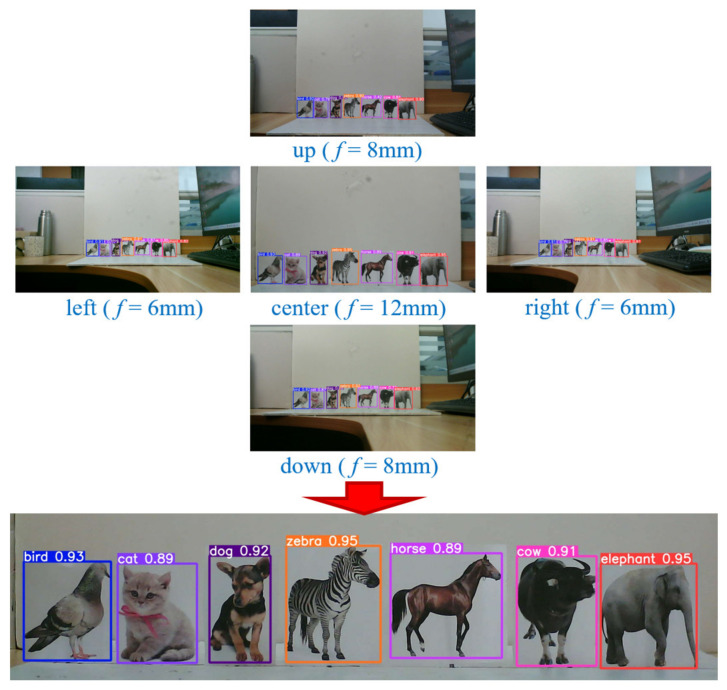
MFBCE target detection results.

To validate the imaging and ranging capabilities of the MFBCE, as well as to assess the robustness of the proposed ranging algorithm, a series of experiments were conducted using the MFBCE system. The experiment captured animal card targets at distances of 100 cm from the MFBCE, as shown in Figure 8. The targets were set as follows: bird, cat, dog, zebra, horse, cow, and elephant. By using the YOLOv5 target detection algorithm, the bounding boxes of the animal card targets were extracted to obtain their coordinates in the pixel coordinate system. Finally, by incorporating pixel size, pixel array, lens focal length, and baseline into the previously derived equations, the distances between the animal card targets and the MFBCE can be calculated. All experimental results are retained to two decimal places.

The MFBCE used in the experiment is configured as described in Section 2. The left and right channels are equipped with 6 mm focal length lenses, the top and bottom channels use 8 mm focal length lenses, and the central channel is fitted with a 12 mm focal length lens. The baseline between the central lens and the peripheral lenses was 19 mm (ΔX/ΔY), while the baseline between the left–right and top–bottom lenses was 38 mm (ΔX/ΔY).

To evaluate the performance of the binocular and multi-eye distance measurement algorithms, we used the following metrics:

MAE (mean absolute error): $\frac{1}{N}\sum_{i=1}^{N}\left|Z_{\mathrm{true}}\left(i\right)-Z_{\mathrm{pred}}(i)\right|$

RMSE (root mean squared error): $\sqrt{\frac{1}{N}\sum_{i=1}^{N}{\left(Z_{\mathrm{true}}\left(i\right)-Z_{\mathrm{pred}}(i)\right)}^{2}}$

### 4.1. Binocular Ranging Experiments Between Lenses with Different Focal Lengths

In this section, we replaced the lenses of the MFBCE to perform binocular ranging experiments. First, we conducted three sets of binocular ranging experiments using lenses with different focal lengths. The baseline distance (ΔX) was set to 38 mm (the baseline distance between the peripheral lenses), and the focal length pairs used in the experiments were 6 mm and 8 mm, 6 mm and 12 mm, and 8 mm and 12 mm. The experimental results are presented in Table 3.

From the results in Table 3, it can be seen that the proposed algorithm successfully enables binocular ranging between cameras with different focal lengths, overcoming the limitations of traditional binocular ranging methods, which typically require identical cameras and prior calibration.

### 4.2. Binocular Ranging Experiments with Different Algorithms

**Figure 9:**
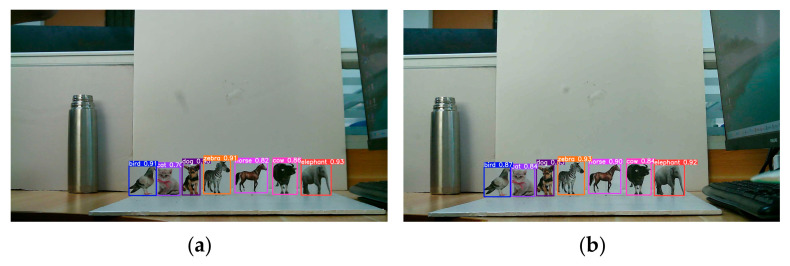
Binocular target detection results. (**a**) Left and (**b**) right.

**Figure 10:**
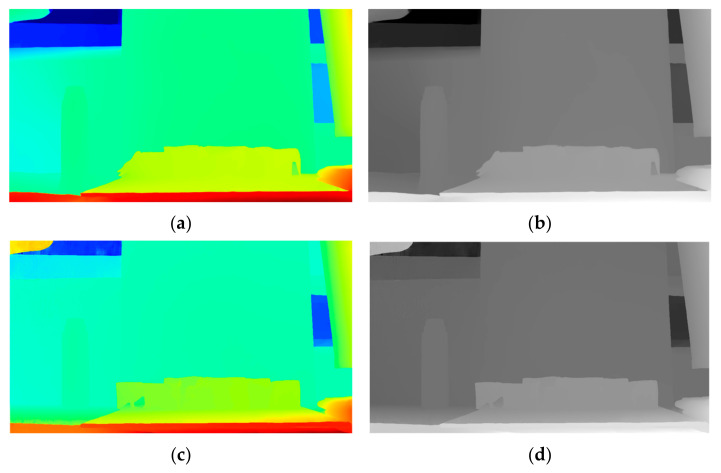
Binocular disparity map. (**a**) RAFT pseudo-color image; (**b**) RAFT grayscale image; (**c**) IGEV++ pseudo-color image; (**d**) IGEV++ grayscale image. The pseudo-color map is mapped in jet format, with red, orange, yellow, green, cyan, blue, and purple colors dis-tributed in order from near to far.

To evaluate the binocular ranging algorithm, we benchmarked our approach against two state-of-the-art algorithms: the widely recognized RAFT-Stereo [34,35] and the recently proposed IGEV++ [36]. Both comparative methods were implemented using their publicly available pre-trained models, which were trained on the standard stereo dataset SceneFlow. The results are presented in Figure 9 and Figure 10, demonstrating the performance of our method relative to these deep learning methods under the same experimental conditions (*f* = 8 mm; Δ*X* = 38 mm).

Subsequently, we incorporated our system’s parameters and, based on the object detection results and disparity maps, computed the ranging accuracy for each method. The experimental results are presented in Table 4.

It is important to note that while RAFT and IGEV++ excel in holistic depth estimation tasks, their architectures inherently prioritize pixel-wise disparity accuracy over object-specific ranging precision—a distinction critical to our application scenario. Our algorithm has higher ranging accuracies for specific objects and does not confuse different targets at the same distance. Furthermore, RAFT and IGEV++ reliance on same-parameter stereo cameras introduces limitations in cross-hardware generalization, a challenge explicitly addressed by our framework.

### 4.3. Binocular Ranging Experiments with Different Object Detection Accuracies

**Figure 11:**
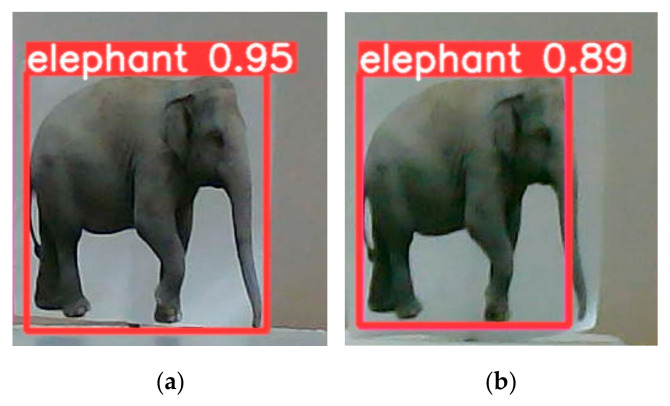
Predicted bounding box offset. (**a**) Normal; (**b**) offset.

In this section, we have supplemented our experiments with an analysis of the impact of object detection accuracy on ranging errors. Figure 11 illustrates cases of bounding box displacement observed during the experiments. Given the complexity of real-world scenarios, we assume no other sources of error (obtain accurate target distance when the predicted bounding box is not offset) and simulate the binocular ranging results under different baseline distances and focal lengths when a single predicted bounding box shifts inward by five pixels. Additionally, we conducted further simulations for a baseline distance of ΔX = 38 mm and a focal length of *f* = 12 mm, analyzing the ranging results when the bounding box shifts by two pixels and one pixel, respectively.

For binocular ranging with identical camera intrinsic parameters and resolution, Equation (18) can be simplified into the following:

(26) $$Z=\frac{f\Delta X}{d(u_{j}-u_{i})}=\frac{f\Delta Y}{d(v_{j}-v_{i})}$$

As shown in Table 5, increasing the baseline distance and focal length reduces the impact of bounding box displacement on ranging errors. Additionally, smaller bounding box shifts result in more accurate ranging outcomes. Therefore, the ranging accuracy can be improved by increasing the focal length and baseline distance, as well as by selecting a higher-precision object detection algorithm to minimize bounding box displacement. In practical applications, higher-precision algorithms typically have slower inference speeds. Considering the trade-off between detection accuracy, processing time, and model deployment on mobile devices, we have chosen the well-established YOLOv5 object detection algorithm. Table 6 shows the performance metrics for various YOLOv5 models trained on the COCO dataset. Finally, we utilize the pre-trained YOLOv5l model to balance performance and efficiency (https://github.com/ultralytics/yolov5, accessed on 20 March 2024).

In the future, with the adaptation of target detection algorithms on mobile platforms such as RV1126, higher-precision object detection algorithms can be employed to further enhance ranging accuracy. Additionally, training the detection model specifically for the target can significantly improve detection precision, thereby leading to more accurate subsequent distance estimation.

### 4.4. Comparison Experiment Between Binocular and Multi-Eye Ranging Algorithms

In this section, we replaced the lenses of the MFBCE to perform binocular ranging experiments. The results of the binocular ranging were then compared to those of the MFBCE’s multi-eye ranging, as shown in Table 7.

Unlike the simulated results in Table 5, which reflect the impact of object detection errors on ranging errors, Table 7 presents the actual ranging results, incorporating both mechanical errors and object detection errors. The results in Table 7 validate our findings in that increasing the baseline distance and using long-focal lenses can effectively reduce the impact of errors and improve ranging accuracy (as discussed in Section 3.3 and Section 4.3). As a result, the binocular ranging algorithm obtains the lowest error (MAE of 1.90 and RMSE of 2.28) when ΔX = 38 mm and f = 12 mm.

**Figure 12:**
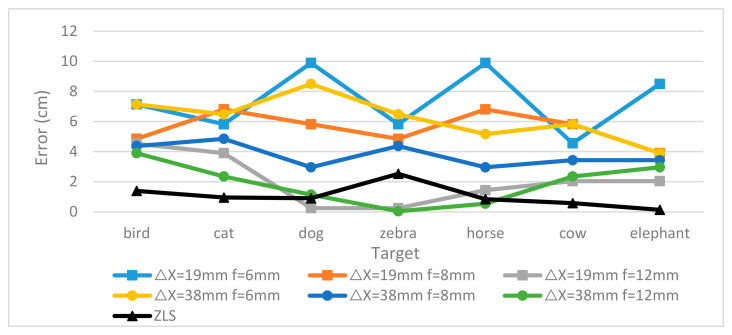
Comparison of absolute errors between binocular and multi-eye ranging (100 cm).

Furthermore, the proposed multi-eye ranging algorithm, based on the MFBCE, effectively integrates information from five different focal length lenses. Without increasing the baseline or using longer focal length lenses, it improves ranging accuracy by directly minimizing the error δ, leading to superior ranging results. The least squares method yielded the smallest error in this approach (MAE of 1.05 and RMSE of 1.26). Figure 12 presents a comparison of the absolute errors between the binocular ranging algorithm and the proposed multi-eye ranging algorithm.

The results presented in Table 8 show the distance estimation performance of the MFBCE system for targets at various distances. It can be observed that as the distance increases, the ranging error of the MFBCE increases significantly. This is because, in order to verify the generalizability of the algorithm, no calibration or registration was performed for the lenses of the MFBCE. The proper registration of a system can significantly reduce mechanical errors, thereby improving both the measurable distance range and overall accuracy.

In summary, the experiments demonstrate the effectiveness of the MFBCE for target detection and distance measurement. The results prove the superiority of the proposed MFBCE target distance measurement algorithm. Compared to traditional binocular ranging methods, the proposed algorithm exhibits strong environmental adaptability, making it suitable for cameras with different focal lengths. Moreover, by effectively integrating multi-eye information, it achieves higher ranging accuracies without the need for calibration.

## 5. Conclusions

This paper presents a multi-focal bionic compound eye (MFBCE) for distance measurements based on multi-eye vision. By utilizing an integrated core board to process image data, the system eliminates the dependence on graphics cards, reducing the weight and volume of the bionic compound eye. By deriving the intrinsic relationship of images obtained with multi-focal-length lenses, this approach overcomes the limitations of stereo and multi-eye ranging algorithms, which require identical cameras and prior calibration. This improvement enhances the system’s distance measurement accuracy and extends its applicability. The experimental results demonstrate that the proposed MFBCE is compact, lightweight, and highly accurate. Combined with the designed multi-eye distance measurement algorithm, it realizes high-precision distance measurements. Without increasing the baseline or using a longer focal length lens, the MAE is one-half of that of the binocular ranging method. This system holds significant potential for applications in areas such as autonomous vehicle obstacle avoidance, robotic grasping, and driver assistance systems.

## Figures and Tables

**Table 1:** System parameters of MFBCE.

Parameter	Typical Value
Size	60 mm × 60 mm × 80 mm
Weight	275 g
Focal length	6 mm, 8 mm, 12 mm
Inter-camera distance (baseline)	19 mm
Optical format	1/2.9 inch
Pixel size	2.8 μm × 2.8 μm
Active pixel array	1920 × 1080

**Table 2:** Field of view of the MFBCE.

Focal Length	DFOV	HFOV	VFOV
6 mm	54.84°	48.26°	28.18°
8 mm	42.52°	37.14°	21.40°
12 mm	29.09°	25.25°	14.36°

**Table 3:** Binocular ranging results of lenses with different focal lengths.

Target	Stereo Δ*X* = 38 mm		
	6 mm and 8 mm	6 mm and 12 mm	8 mm and 12 mm
bird	100.31	101.27	101.00
cat	104.92	101.07	100.56
dog	103.90	95.24	101.66
zebra	107.74	102.97	106.10
horse	97.86	98.95	102.49
cow	98.16	96.74	101.81
elephant	101.27	93.78	105.69
MAE	3.16	2.94	2.76
RMSE	3.95	3.48	3.44

**Table 4:** Binocular ranging results with different algorithms.

Target	Stereo ΔX = 38 mm, *f* = 8 mm		
	RAFT	IGEV++	Ours (Detection)
bird	111.69	107.20	105.09
cat	110.54	106.67	105.09
dog	110.54	106.14	103.56
zebra	109.97	105.61	104.07
horse	109.40	105.61	102.56
cow	109.40	105.61	103.56
elephant	108.84	104.58	102.07
MAE	10.05	5.92	3.71
RMSE	10.09	5.97	3.86

**Table 5:** Simulated binocular ranging results with predicted bounding box offsets.

Distance (cm)	Stereo ΔX = 19 mm			Stereo ΔX = 38 mm				
	6 mm	8 mm	12 mm	6 mm	8 mm	12 mm	12 mm 2 Pixel	12 mm 1 Pixel
90	101.18	98.13	95.26	95.26	93.89	92.56	91.01	90.50
95	107.55	104.11	100.88	100.88	99.35	97.85	96.21	95.56
100	114.00	110.14	106.54	106.54	104.83	103.17	101.24	100.62
105	120.54	116.24	112.24	112.24	110.34	108.50	106.37	105.68
110	127.18	122.40	117.97	117.97	115.87	113.84	111.51	110.75
115	133.91	128.62	123.74	123.74	121.43	119.21	116.65	115.82
120	140.74	134.91	129.55	129.55	127.02	124.59	121.79	120.89

**Table 6:** Performance metrics for various YOLOv5 models.

Model	Size	mAP^val^ 50–95	mAP^val^ 50	Speed CPU b1 (ms)	Speed RV1126 (ms)	Params (M)
YOLOv5n	640	28.0	45.7	45	33	1.9
YOLOv5s	640	37.4	56.8	98	65	7.2
YOLOv5m	640	45.4	64.1	224	144	21.2
YOLOv5l	640	49.0	67.3	430	280	46.5
YOLOv5x	640	50.7	68.9	766	502	86.7

**Table 7:** MFBCE binocular and multi-eye ranging results (100 cm).

Target	Stereo ΔX = 19 mm			Stereo ΔX = 38 mm			Ours (MFBCE)		
	6 mm	8 mm	12 mm	6 mm	8 mm	12 mm	Z_AM_	Z_RMS_	Z_LS_
bird	107.14	104.85	104.53	107.14	104.37	103.90	102.52	102.53	101.39
cat	105.82	106.81	103.90	106.48	104.85	102.35	101.06	101.07	100.95
dog	109.89	105.82	100.25	108.50	102.96	101.14	101.11	101.15	100.90
zebra	105.82	104.85	100.25	106.48	104.37	99.96	103.85	103.86	102.53
horse	109.89	106.81	101.44	105.17	102.96	100.54	100.99	101.01	100.84
cow	104.56	105.82	102.04	105.82	103.43	102.35	99.49	99.50	99.42
elephant	108.50	103.90	102.04	103.90	103.43	102.96	99.94	99.98	99.86
MAE	7.37	5.55	2.06	6.21	3.77	**1.90**	1.44	1.45	**1.05**
RMSE	7.63	5.64	2.57	6.36	3.83	**2.28**	1.88	1.89	**1.26**

**Table 8:** MFBCE ranging results (different distances).

Target	Ours (MFBCE) Z_LS_					
	100 cm	120 cm	140 cm	160 cm	180 cm	200 cm
bird	101.39	121.77	144.22	172.89	193.07	218.60
cat	100.95	123.21	144.71	170.44	193.72	215.56
dog	100.90	123.60	147.20	169.00	195.22	216.10
zebra	102.53	122.94	147.27	171.00	192.91	214.67
horse	100.84	122.13	144.83	169.94	190.07	213.12
cow	99.42	122.06	144.97	168.24	193.03	210.84
elephant	99.86	121.34	144.54	169.44	185.09	209.53
MAE	1.05	2.43	5.39	10.15	11.87	14.03
RMSE	1.26	2.55	5.52	10.25	12.27	14.34

## Data Availability

Data are contained within the article.
